# Prefrontal cortical dopamine deficit may cause impaired glucose metabolism in schizophrenia

**DOI:** 10.1038/s41398-024-02800-7

**Published:** 2024-02-06

**Authors:** Qiongqiong Wu, Yujun Long, Xingjie Peng, Chuhan Song, Jingmei Xiao, Xiaoyi Wang, Furu Liu, Peng Xie, Jinqing Yang, Zhe Shi, Zhonghua Hu, Colin McCaig, David St Clair, Bing Lang, Renrong Wu

**Affiliations:** 1 National Clinical Research Center for Mental Disorders, and Department of Psychiatry, The Second Xiangya Hospital of Central South University, Changsha, 410011 Hunan China; 2 Affiliated Mental Health Centre & Hangzhou Seventh People’s Hospital, Zhejiang University School of Medicine, Hangzhou, Zhejiang 310013 China; 3 Key Laboratory for Quality Evaluation of Bulk Herbs of Hunan Province, Hunan University of Chinese Medicine, Changsha, Hunan China; 4 Hunan Key Laboratory of Molecular Precision Medicine, Department of Critical Care Medicine, Xiangya Hospital, Central South University, Changsha, Hunan 410008 China; 5 School of Medical Sciences, Institute of Medical Sciences, University of Aberdeen, Foresterhill, Aberdeen, AB25 2ZD UK

**Keywords:** Pathogenesis, Neuroscience

## Abstract

The brain neurotramsmitter dopamine may play an important role in modulating systemic glucose homeostasis. In seven hundred and four drug- naïve patients with first-episode schizophrenia, we provide robust evidence of positive associations between negative symptoms of schizophrenia and high fasting blood glucose. We then show that glucose metabolism and negative symptoms are improved when intermittent theta burst stimulation (iTBS) on prefrontal cortex (PFC) is performed in patients with predominantly negative symptoms of schizophrenia. These findings led us to hypothesize that the prefrontal cortical dopamine deficit, which is known to be associated with negative symptoms, may be responsible for abnormal glucose metabolism in schizophrenia. To explore this, we optogenetically and chemogenetically inhibited the ventral tegmental area (VTA)-medial prefrontal cortex (mPFC) dopamine projection in mice and found both procedures caused glucose intolerance. Moreover, microinjection of dopamine two receptor (D2R) neuron antagonists into mPFC in mice significantly impaired glucose tolerance. Finally, a transgenic mouse model of psychosis named Disc1_tr_ exhibited depressive-like symptoms, impaired glucose homeostasis, and compared to wild type littermates reduced D2R expression in prefrontal cortex.

## Introduction

Patients with schizophrenia present with positive symptoms (delusions, hallucinations, disorganized thinking or speech), negative symptoms (diminished expression and motivation). The dopamine hypothesis of schizophrenia proposes that positive symptoms mainly result from hyperfunction of dopaminergic mesolimbic projections, and negative symptoms mainly result from deficits in dopamine transmission in mesocortical pathways [1]. There is robust evidence for prefrontal cortical hypodopaminergia in schizophrenia. For example, immunocytochemical studies of postmortem specimens showed that the dopamine innervation of the prefrontal cortex in the brains of patients with schizophrenia was reduced [2]. Positron emission computed tomography (PET) studies using Dopamine 1 receptor (D1R) or Dopamine 2 receptor (D2R) radioligand have found patients with schizophrenia had a reduction in D1R and D2R availability in prefrontal cortex (PFC), especially in dorsolateral prefrontal cortex (DLPFC) [3–6]. Negative symptoms in schizophrenia were associated with dopamine deficits in prefrontal cortex via PET dopamine imaging studies [5, 6]. In addition to this clinical evidence, there are some principal rodent studies linking negative symptoms to prefrontal cortical dopamine. In rodent studies, negative symptoms, included in *Negative Valence Systems and Systems for Social Processes* in Research Domain Criteria (RDoc), are mostly classified into depressive-like phenotype in behavior tests (https://www.nimh.nih.gov/research/research-funded-by-nimh/rdoc/constructs/rdoc-matrix). Genetic models of schizophrenia together with environmental stress models, such as dominant-negative DISC1 (Disrupted-in-schizophrenia-1) transgenic mouse plus 3-week isolation stress, showed remarkable depressive-like symptoms and decreased dopamine in PFC [7]. The depressive-like phenotype was regulated by VTA-mPFC dopamine projection, whose inhibition increased susceptibility to social-defeat stress [8].

Disorders in glucose metabolism are common in schizophrenia. The rate of type 2 diabetes (T2DM) in schizophrenia is 3-4 times that of the general population [9], and it partly accounts for decreased life expectancy of patients with schizophrenia [10, 11]. However, even drug-naïve patients with first episode schizophrenia, where the confounding effects of the course of disease and antipsychotic drugs can be excluded, have increased risk of impaired glucose homeostasis [11, 12]. Meta-analyses shows that patients with schizophrenia have higher fasting plasma glucose, higher fasting plasma insulin, higher insulin resistance (HOMA-IR) and impaired glucose tolerance compared to controls [11–13]. However, our own studies have found that impaired glucose homeostasis was rare in patients with severe positive symptoms [14, 15]. Others have also found that positive symptoms of schizophrenia are negatively associated with fasting glucose and HOMA-IR [16, 17]. We concluded that glucose homeostasis varies in different kinds of patients with schizophrenia, and negative symptoms are more likely to be accompanied by impaired glucose homeostasis. However, the mechanisms responsible for early derangements of glucose homeostasis in schizophrenia have not to date been elucidated [11].

Dopamine in brain may regulate systemic glucose metabolism. It has been reported that obese groups tend to have lower striatal D2/3 R availability compared with lean groups, and this is in proportion to their BMI [18]. Deep brain stimulation (DBS) which aims to increase striatal dopamine increases the insulin sensitivity of peripheral tissue [19]. Conversely, the systemic depletion of dopamine via a-methyl-para-tyrosine (AMPT) impaired peripheral insulin–mediated glucose uptake [19]. What is more, a PET study reported that extracellular dopamine levels in VTA were high when plasma glucose levels were low in healthy volunteers, but this correlation was disrupted in patients with schizophrenia [20].

In this study, we first examined the relationship between glucose metabolism and negative symptoms in a cross-sectional study of drug naïve first episode patients with schizophrenia. Secondly, we treated patients with predominant negative symptoms with intermittent theta-burst stimulation (iTBS) on dorsolateral prefrontal cortex (DLPFC) to explore whether glucose metabolisms can be improved via activating DLPFC. Thirdly, we determined whether impairing dopamine projection from VTA to mPFC results in glucose intolerance via optogenetics, chemogenetics and microinjections of antagonists in mice. Finally, we examined Disc1_tr_ mice for evidence of PFC dopamine defects and impaired glucose homeostasis. In summary, we aimed to determine whether the VTA-PFC dopamine projection may be responsible for abnormal glucose metabolism in schizophrenia.

## Materials and methods

### Patients

In this cross-sectional study, a total of 704, drug naïve patients diagnosed with schizophrenia based on DSM-IV Structured Clinical Interview were included. The sample size for a two-tailed, effect-size = 0.3, *α* = 0.05, power = 0.8 analysis is 80. Thus, the number of included patients was sufficient. Patients with symptoms of over 60 months duration were excluded from this database according to previous studies [11, 12]. Patients with schizophrenia provided serum and plasma samples after an overnight fast. Glucometabolic parameters (fasting plasma glucose/FG, fasting plasma insulin/FI) and lipid profiles (total cholesterol/Chol, triglycerides/TG, high-density lipoprotein cholesterol/HDL-C and low-density lipoprotein cholesterol/LDL-C).

The transcranial magnetic stimulation (TMS) human study is a real-world naturalistic prospective study from Jan. 2021 to Dec. 2022. This study aimed to detect a between-group difference of at least 6 points in SANS scores, and we calculated that 25 patients per group were required based on previous studies [21] (*α* = 0.05, power = 0.8). Thus, we aimed to recruit 60 patients at 15% dropout rate. A total of 57 chronic patients with predominant negative symptoms (Positive and Negative Syndrome Scale factor scores for negative symptoms were 20 or more and positive symptom scores were 19 or less) [22] were recruited from the Department of Psychiatry, the Second Xiangya Hospital. These patients were diagnosed with schizophrenia according to DSM-5, and they had steady medical condition and antipsychotic medication during the TMS intervention. Exclusion criteria were psychiatric comorbidities, use of antidepressants or mood stabilizer, organic brain diseases or drug abuse/dependence. In this study, 28 patients received iTBS, and 29 patients matched with age, duration of illness and clinical symptoms (PANSS scores) were collected in Control group. The negative symptoms of these patients were assessed via Scale for the Assessment of Negative Symptoms (SANS) and PANSS. The SANS scores and blood sample of both groups were collected at baseline/pre-treatment and Day 11/post-treatment. The primary outcome was the change of SANS between pre-treatment and post-treatment, and the secondary outcome was the change of glucometabolic parameters.

In these two human studies, PANSS and SANS were assessed by trained psychiatrists.

Insulin resistance was calculated by the homeostasis model assessment of insulin resistance: HOMA-IR = fasting plasma glucose (mmol/l) × fasting plasma insulin (μU/ml)/22.5. According to the American Diabetes Association, the cutoff value for impaired fasting glucose is 5.6 mmol/l for fasting plasma glucose (American Diabetes Association, 2010) and 2.5 for HOMA-IR [23]. The cross-sectional study was reported according to STROBE statement reporting guidelines.

### iTBS intervention

Before the intermittent theta-burst stimulation (iTBS) intervention, patients in the iTBS group had magnetic resonance imaging (MRI) scan. To direct and target the iTBS location, we used the anatomical T1-weighted images and neuronavigation system (localite TMS Navigator), and then we identified stereotactic scalp coordinates overlying the left dorsolateral superior frontal gyrus (MNI coordinate, *X* = −18.45 mm, *Y* = 34.81 mm, *Z* = 42.20 mm). The patients in iTBS group received iTBS for 10 consecutive days and 3 times per day in a quiet therapy room. The interval between each intervention was at least 30 minutes. The stimuli were delivered via the MagPro X100 stimulator (MagVenture) and a standard figure 8 coil (MagVenture Cool-B65). The iTBS treatment had an intensity of 80% of the individual resting motor threshold (MT), and the other parameters were set to triplet 50 Hz bursts, repeat at 5 Hz, 2 s on and 8 s off, 600 pulses per session, total duration of 3 min and 9 s. The iTBS treatment was performed by the specialized operators.

### Animals

C57BL/6 male mice were purchased from Hunan SJA Laboratory Animal Co., Ltd. Disc1_tr_ were kindly provided by University of Aberdeen. Disc1_tr_ transgenic mice expressed 2 copies of truncated Disc1, which is associated with is associated with schizophrenia. DAT-Cre mice (Jackson Laboratory #006660) were kindly provided from Ji Hu Lab, School of Life Science and Technology, ShanghaiTech University. The animals’ care was in accordance with guidelines of the Animal Advisory Committee of Central South University.

### Stereotaxic surgeries

Before the surgery, mice were anesthetized and head-fixed in a stereotaxic frame. For optogenetic inhibition, the AAVs (AAV2/9-hEF1a-DIO-eNpHR 3.0-mCherry-WPRE-pA, AAV2/9-hEF1a-DIO-mCherry-WPRE-pA, Taitool Bioscience (Shanghai)) were injected intracranially into the bilateral VTA (bregma -3.2 mm, lateral ±0.4 mm, ventral 4.2 mm) of 8-week-old DAT-Cre mice. Optical fibers were fixed bilaterally mPFC (bregma 1.7 mm, lateral 0.65 mm, ventral 2.1 mm) and fixed to the skull with dental cement. For chemogenetic inhibition, we injected AAV2/retro pAAV-TH-Cre-P2A-EGFP-WPRE (Obio Technology (Shanghai) Corp., Ltd) into the mPFC and injected rAAV-hSyn-DIO-hM4D(Gi)-mCherry-WPRE-hGH polyA or rAAV-hSyn-DIO-mCherry-WPRE-hGH polyA (Brain-VTA, Wuhan) in the VTA. All experiments were performed 3–4 weeks after viral injection.

### Optogenetic inhibition

The eNpHR3.0 (halorhodopsin) achieved light-triggered inhibition of neural activity via pumping into neurons an ion (chloride). The mice were tested four weeks after surgeries to allow the full expression of eNpHR3.0. An ultra-high-power LED (589 nm, QAXK-LASER-589, Thinker Tech Nanjing Bioscience Inc.) was used for optical stimulation (output intensity of 6–10 mW, continuous stimulation at 590 nm for eNpHR3.0). Mice were given a protocol of 30 s of yellow light on and then 5 s light off during the glucose tolerance test. The mice habituated to fiber connection at least three days before test.

### Chemogenetic inhibition

The designer receptors exclusively activated by designer drugs (DREADDs) was developed by Armbruster et al. [24, 25]. The hM4Di, the most used inhibitory DREADD, can be activated by CNO. The mice in the hM4D(Gi) group and Vehicle group were intraperitoneally given 0.33 mg/ml CNO (the clozapine-N-oxide, 3.3 mg/kg). After CNO injection, the glucose tolerance test was immediately started.

### Dopamine receptor inhibitor microinjection

Mice were head-fixed and then after anesthesia via isoflurane, they received bilateral intracranial injections of the D1/D2 receptor inhibitors. Guides were placed in mPFC at least a week before injection. Then, a double cannula coupled to syringes and a dual-syringe infusion pump, extending 0.2 mm below the guides. Drugs or vehicle were injected at a rate of 0.3 μL/min, and the cannulas were left in place for 2 min. Doses were SCH23390 (D1R inhibitor, Sigma-Aldrich) and raclopride (D2R inhibitor, MedChemExpress) in 0.6 μL per hemisphere. All tests were performed 30–60 min after injection.

### Behavioral test

All behavioral tests were performed on mice at 8–14 weeks in the light phase. Before every test, mice were habituated in the testing room and the wearing in optogenetic tests. All tests were performed by researchers blinded to the genotype.

### Open field test

A clear plexiglas box (33 × 33 × 33 cm) was used for the open field task. Mice were introduced into the center of the chamber and allowed to explore for 5 min. The movements of mice in open field test, location recognition and temporal order recognition were all recorded and analyzed by TopScan (CleverSys Inc, Reston, VA, USA). Locomotor activity was evaluated via total distance and anxiety-like behavior was evaluated via time in the center of open field test.

### Elevated plus maze

The EPM apparatus has two open arms (30 × 5 cm) and two closed arms (30 × 5 × 20 cm) elevated 50 cm above the floor. At the beginning of the experiments, mice were placed in the center facing the open arms and back to the operator. They were allowed to explore for 5 min. Anxiety-like behavior was evaluated via the time spent in the open arms.

### Tail suspension test

Tail suspension test was conducted in three-walled boxes (50 cm × 40 cm × 20 cm). The tails of mice were adhered to a bar by tape (1 cm from tail tip). Mice were suspended roughly 20 cm above the apparatus floor for 6 min. The immobility time was calculated with investigators blinded to the group or genotype. Depression-like behavior was evaluated via the immobility time.

### Three-chambered test

The mice were habituated to 3-chamber apparatus containing two empty wire cages. The test had 2 phases: (A) Pre-test (Phase 1): two identical paper balls (NS1 and NS2) were placed in the two cages. The mouse was placed into the center chamber and freely explored for 10 min. (B) Sociability (Phase 2): an age- and gender-matched stranger wild-type mouse (S1) was placed in one of a wire cage; a novel non-social stimulus was placed in the other cage. The close interaction time with the cages in these two phases were measured and analyzed.

### Intraperitoneal glucose tolerance test (IGTT)

Mice were adapted for intraperitoneal injection by handling and sham intraperitoneal injection at least three days before test. After overnight fasting from 18:00 to 9:00, mice were transported to a testing room and individually tethered to an overhead fiber-optic cable via their indwelling optic fibers. They were allowed to move freely. A glucose dose of 2 g/kg was injected intraperitoneally. Immediately upon injection, the laser was turned on (a repeating profile of 3 min on, 10 s off) for the duration of the test in optogenetic inhibition. For chemogenetic inhibition, 0.33 mg/ml CNO (the clozapine-N-oxide, 3.3 mg/kg) was intraperitoneally given injected immediately before the IGTT. Blood glucose levels at 15, 30, 60, 90 and 120 min after injection were monitored using the glucometer (Roche). Calculation of the area under the curve (AUC) above baseline was conducted to validate the results.

### Western blot

Mice were anesthetized and sacrificed, and then their brains were disserted. The PFC of mice was dissected out according to the atlas. Isolated brain tissue was homogenized in RIPA buffer (sigma) with complete protease inhibitor mixture (Roche) on ice and centrifuged to remove large debris. The protein concentration of the supernatant was determined using bicinchoninic acid (BCA) protein assay. Protein was resolved by electrophoresis on SDS-PAGE gels and transferred onto polyvinylidene difluoride membranes (PVDF; Millipore). The membranes were blocked in 5% nonfat milk in Tris-buffered saline with Tween for 1 h and were incubated with the following primary antibodies overnight at 4 °C: anti-TH (sigma), anti-dopamine D1 receptor (D1R) (Thermo Fisher scientific), anti-dopamine D2 receptor (D2R) (Santa) antibodies were used as primary antibodies. After several washes with Tris-buffered saline with Tween, the blots were incubated in horseradish peroxidase-conjugated secondary antibody (sigma) at 1:3000 for 1 h. After several washes with Tris-buffered saline with Tween, immunoreactive antibody–antigen complexes were visualized using the Immobilon forte Western HRP Substrate (Millipore) by Amersham™ ImageQuant™ 800.

### RT-PCR

Total RNA was extracted from brain tissue with TRIzol, and RNA sample was converted to cDNA via the PrimeScriptTM RT reagent Kit (Roche). RT-PCR was performed in triplicate for each sample with SYBR Green Premix Taq (Roche). The primer sequences were: D2R (Forward ACCTGTCCTGGTACGATGATG; Reverse GCATGGCATAGTAGTTGTAGTGG).

### Statistics

The missing values in the database of two clinical studies were imputed via multiple imputation (MI) in R studio. The assumption of normality for continuous variables was tested using the Kolmogorov–Smirnov/Shapiro–Wilks test. We used ANOVA for normally distributed continuous values and the Mann–Whitney *U* test for skewed continuous variables. Categorical variables were analyzed with the *χ*^2^ test or Fisher exact test. Effect sizes were calculated as Cohen *d* for continuous outcomes. Spearman correlation analysis was performed to investigate glucose metabolic parameters and clinical symptoms. *P*-value was corrected via Bonferroni correction if multiple comparisons were made. All analyses were performed via R 4.2.1 and SPSS23.0.

### Study approval

In human studies, we received written informed consent prior to participation. All these human studies and rodent studies were reviewed and approved by the ethics committee of Second Xiangya Hospital, Central South University.

## Results

### Abnormal glucose metabolism is highly associated with negative symptoms in patients with schizophrenia

A total of 705 drug-naïve patients from Feb 2012 to Dec 2021 diagnosed with schizophrenia according to DSM-IV Structured Clinical Interview was potentially eligible for this study. One patient whose duration of illness over 60 months was excluded, and 704 patients were included in this study. In our collected data, the average age, duration of symptoms, BMI of these patients were presented in Table S1. Their clinical characteristics were: PANSS total score was 81.82 ± 17.43; PANSS positive, negative, and general psychopathology scores were 20.81 ± 6.77, 20.75 ± 7.38 and 40.31 ± 9.43, respectively (see Table S1). There were 34 missing FGs and 78 missing FIs imputed via multiple imputation (MI) (Table 1). The relationships between glucose metabolism parameters and clinical symptoms in patients are shown in Table 2. There were robust associations between glucose metabolism parameters and Positive and Negative Syndrome Scale factor score for negative symptoms (PANSS-N). Higher FG, higher FI and higher HOMA-IR were significantly associated with higher PANSS-N: *r*_(FG, PANSS-N)_ = 0.229, *P* < 0.0001; r _(FI,PANSS-N)_ = 0.221, *P* < 0.0001; *r*_(HOMA-IR,PANSS-N)_ = 0.259, *P* < 0.0001, see Table 1). Besides, there was a positive association between HOMA-IR and general psychopathology subscale (*r* = 0.150, *P* = 0.032). The Correlation Test Matrices of all these parameters are shown in Table S3.

**Table 1:** Correlations of glucose parameters and other characteristics.

	FG		FI		HOMA-IR	
	*r*	Adjusted-*P*	*r*	Adjusted-*P*	*r*	Adjusted-*P*
Age (years)	0.058	1.000	−0.030	1.000	−0.015	1.000
Duration of illness (months)	0.116	0.067	0.080	1.000	0.102	0.232
PANSS						
Positive score	−0.005	1.000	0.030	1.000	0.030	1.000
Negative score	**0.229**	<0.0001*	**0.221**	**<0.0001**	**0.259**	**<0.0001**
General psychopathology	0.106	0.164	0.105	0.171	**0.150**	**0.032**
Total score	**0.151**	0.002*	**0.173**	**<0.001**	**0.198**	**<0.0001**
TG (mmol/L)	0.107	0.146	−0.012	1.000	0.006	1.000
Chol (mmol/L)	0.116	0.067	0.013	1.000	0.034	1.000
HDL-C (mmol/L)	0.025	1.000	−0.005	1.000	0.003	1.000
LDL-C (mmol/L)	0.073	1.000	−0.018	1.000	−0.006	1.000

Adjusted-P: corrected *P*-value via Bonferroni correction.

*FG* fasting glucose, *FI* fasting insulin, *TG* Triglyceride, *Chol* Total cholesterol, *HDL-C* High-density lipoprotein cholesterol, *LDL-C* Low-density lipoprotein cholesterol, *PANSS* Positive and Negative Syndrome Scale.

*P < 0.05.

The significant values are highlighted in Bold.

Blind to outcome measures, we retrospectively selected patients with predominant negative symptoms if PANSS-N were 20 or more and positive symptom scores were 19 or less [22]. Abnormal rates of fasting glucose in patients with predominant negative symptoms were significantly higher than the other patients (66/209 vs. 96/495, *P* < 0.001). Next, also blind to outcome measures, we divided patients into 4 groups: (a) patients with predominant negative symptoms/ Negative group, (b) patients with major positive symptoms/ Positive group (positive symptom scores were 20 or more and negative symptoms were 20 or less), (c) patients with both severe negative symptoms and positive symptoms/Negative&Positive group (if positive and negative symptom scores were both 20 or more), and (d) other patients. The four groups comprised 209, 178, 202 and 115 subjects, respectively. The characteristics of these four groups are shown in Table 2. The duration of symptoms, and sex of these groups were not matched. As expected, the mean fasting glucose in Negative group was significantly higher than Positive group (*P* = 0.011). The abnormal rates of fasting glucose in Negative group were significantly higher than Positive group (66/209 vs. 24/178, *P* < 0.001), while there was no significant difference in BMIs between these groups. All these results highlighted the robust relationship between negative symptoms and impaired glucose metabolism.

**Table 2:** Characteristics of four groups in 704 first-episode patients with schizophrenia.

	Negative group (*n* = 209)	Positive group (*n* = 178)	Negative & Positive group (*n* = 202)	Other patients (*n* = 115)	*P*^*a*^	Negative *vs*. Positive *P*^b^	Negative *vs*. Negative & Positvie *P*^b^
Age	26.33 (6.87)	29.61 (10.65)	27.05 (8.89)	26.80 (8.89)	0.035*	0.111	1.000
Sex (Male, Female)	(119, 90)	(68, 110)	(94, 108)	(55, 60)			
Duration of illness (months)	20.65 (14.66)	10.78 (11.21)	13.05 (10.66)	13.63 (12.59)	<0.001*	<0.001*	<0.001*
BMI	21.66 (3.52)	21.44 (3.55)	21.43 (4.36)	22.14 (4.18)	0.296		
PANSS							
Positive score	15.01 (2.79)	25.91 (4.57)	25.79 (4.83)	14.72 (3.31)	<0.001*	<0.001*	<0.001*
Negative score	26.18 (4.47)	13.65 (3.44)	25.48 (4.76)	13.54 (3.95)	<0.001*	<0.001*	0.510
General psychopathology	39.93 (6.43)	40.04 (8.00)	46.64 (9.96)	31.36 (6.77)	<0.001*	1.000	<0.001*
Total score	80.42 (9.95)	79.51 (11.16)	97.85 (15.53)	59.77 (10.87)	<0.001*	1.000	<0.001*
FG (mmol/L)	5.30 (1.11)	4.89 (0.70)	5.29 (1.04)	4.87 (0.64)	0.001*	0.011*	1.000
FG abnormal rate	66/209 (31.57%)	24/178 (13.49%)	64/202 (31.68%)	8/115 (6.96%)		<0.001*	0.982
FI (mU/L)	18.03 (12.87)	13.92 (7.53)	18.51 (12.55)	14.25 (8.37)	0.002*	0.011*	1.000
HOMA-IR	4.47 (3.69)	3.02 (1.69)	4.52 (3.46)	3.13 (2.03)	<0.001*	0.014*	1.000
HOAM-IRabnormal rate	128/209 (61.24%)	92/178 (51.69%)	133/202 (65.84%)	57/115 (49.57%)		0.058	0.333
TG (mmol/L)	1.30 (0.89)	1.20 (0.93)	1.33 (1.19)	1.23 (1.00)	0.489		
Chol (mmol/L)	4.30 (2.85)	4.12 (1.07)	4.04 (1.09)	4.18 (0.99)	0.774		
HDL-C (mmol/L)	1.25 (0.41)	1.33 (0.37)	1.34 (0.60)	1.30 (0.43)	0.021*	0.012*	0.425
LDL-C (mmol/L)	2.31 (0.64)	2.53 (0.73)	2.44 (0.66)	2.47 (0.74)	0.074		

Values are presented as mean (SD).

*PANSS* Positive and Negative Syndrome Scale, *FG* fasting glucose, *FI* fasting insulin, *TG* triglyceride, *Chol* total cholesterol, *HDL-C* high-density lipoprotein cholesterol, *LDL-C*, low-density lipoprotein cholesterol.

*P < 0.05.

*P*^*a*^: *P*-value for the overall differences among four groups was tested via Kruskal–Wallis test.

*P*^b^: Follow-up pairwise comparisons were performed via Kruskal–Wallis test.

The significant values are highlighted in Bold.

### Theta-burst stimulation of DLPFC improved negative symptoms and glucose metabolisms in patients with negative symptoms

Intermittent theta-burst stimulation (iTBS), a protocol of repetitive transcranial magnetic stimulation is a new intervention to improve negative symptoms in schizophrenia by temporarily exciting specific areas in the brain [26–28]. It has potential to enhance dopaminergic signaling [29], and iTBS of PFC improves the negative symptoms by reversing a hypodopaminergic state in PFC [30, 31]. To further investigate the associations between negative symptoms and glucose metabolism in patients with schizophrenia, we recruited 60 patients with predominant negative symptoms in Department of Psychiatry, the Second Xiangya Hospital, from Jan. 2021 to Dec. 2022. A total of 57 patients finished the study (Participant flow see Table S4). One group underwent iTBS (*n* = 28) while the matched Control group (*n* = 29) did not receive iTBS, and both groups had steady antipsychotic treatment regimen during this trial. These two groups had similar distribution of antipsychotic use (iTBS/Control): amisulpride 9/8, olanzapine 6/5, risperidone 11/9, aripiprazole 2/6, others 1/1. The demographic, baseline clinical characteristics and glucometabolic parameters were shown in Table S2.

The negative symptoms assessed via Scale for Assessing Negative Symptoms (SANS) and mean glucometabolic parameters at pre-treatment and eleven days post-treatment timepoint as well as ∆ of pre- and post-treatment are presented in Table 3. In the repeated-measures ANOVA for negative symptoms, the time main effect (*F* = 20.762, *P* < 0.001) and the interaction between group and time (F = 17.409, *P* < 0.001) were significant. In the intention-to-treat analysis, the iTBS group was superior to Control when comparing the change in glucometabolic parameters and SANS scores. The mean difference of SANS and FG are −12.73 (95% CI, −18.85 to −6.62; *P* < 0.001) and -0.54 (95% CI, −0.9 to −0.18; *P* = 0.004). These results indicate that iTBS can not only improve negative symptoms but also decrease FG. Of note, there were positive associations between the change in glucometabolic parameters and the change in SANS scores: The change in SANS score is positively associated with the change in FI (*r* = 0.464, *P* < 0.001) and HOMA-IR (*r* = 0.502, *P* < 0.001). In conclusion, iTBS targeted at activating dopaminergic circuits, improved glucose metabolisms in patients with predominant negative symptoms.

**Table 3:** The effects of iTBS on SANS and glucometabolic parameters in patients with schizophrenia.

	Pre-treatment		Post-treatment		Repeated measure test within group (*P* value)		∆ of pre- and post-treatment				
	iTBS	Control	iTBS	Control	iTBS	Control	iTBS	Control	Mean difference (95%CI)	Cohen *d* (95%CI)	*P* value
SANS	68.32 (11.84)	66.38 (7.12)	55 (16.76)	65.79 (8.91)	0.001	0.077	−13.32 (15.29)	-0.59 (5.94)	−12.73 (−18.85 to −6.62)	1.11 (0.54–1.66)	<0.001*
FG (mmol/L)	5.52 (0.84)	5.19 (0.79)	5.16 (1.01)	5.37 (1.00)	0.181	0.720	−0.36 (0.78)	0.18 (0.57)	−0.54 (−0.9 to −0.18)	0.79 (0.25–1.33)	0.004*
FI (mU/L)	13.28 (7.55)	12.40 (6.86)	9.63 (4.84)	13.99 (6.35)	0.063	0.231	−3.65 (7.11)	1.59 (8.07)	−5.24 (−9.28 to −1.19)	0.69 (0.15–1.22)	0.012*
HOMA-IR	3.34 (2.14)	2.91 (1.89)	2.15 (0.99)	3.33 (1.56)	0.046	0.211	−1.19 (1.97)	0.41 (2.07)	−1.60 (−2.68 to −0.53)	0.79 (0.25–1.33)	0.004*

Values are presented as mean (SD). iTBS group underwent active iTBS while Control group underwent sham stimulation.

*FG* fasting glucose, *FI* fasting insulin, *SANS* Scale for the Assessment of Negative Symptoms, *∆ of pre- and post-treatment* change from pre-treatment to post-treatment.

*P < 0.05.

The significant values are highlighted in Bold.

### Optogenetic inhibition of VTA-mPFC dopamine projection caused glucose intolerance in mice

**Fig. 1:**
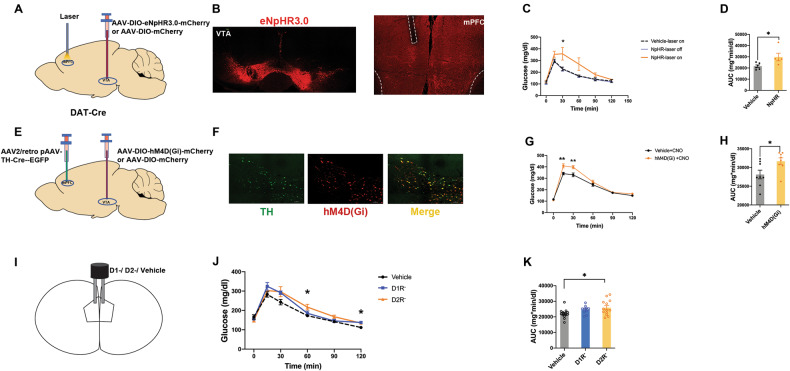
Inhibition of VTA-mPFC dopamine projection or blocking dopamine receptors in mPFC resulted in glucose intolerance. **A** Schematic experimental strategy for optogenetic inhibition the VTA-mPFC dopamine pathway in DAT-Cre mice. **B** Confocal image showing AAV2/9-hEF1a-DIO-eNpHR 3.0-mCherry-WPRE-pA in VTA and eNpHR 3.0 terminals and fiber optic cannula placements in mPFC. **C**, **D** Glucose tolerance of NpHR groups with laser-on, vehicle groups with laser-on, NpHR groups with laser-off (The comparison was only made between NpHR+laser-on and vehicle+laser-on. 0 min: F_1,9_ = 0.186, *P* = 0.676; 15 min: F_1,9_ = 3.829, *P* = 0.082; 30 min: F_1,9_ = 6.262, *P* = 0.034; 60 min: F_1,9_ = 4.249, *P* = 0.069; 90 min: F_1,9_ = 4.011, *P* = 0.076; 120 min: F_1,9_ = 0.605, *P* = 0.457; n = 5-6). AUC, area under curves in glucose curve to present glucose tolerance (F_1,9_ = 6.035, *P* = 0.036; *n* = 5-6). **E** Schematic experimental strategy to chemogenetic inhibition the VTA-mPFC dopamine projection. **F** Confocal image showing AAV2/retro pAAV-TH-Cre and hM4D(Gi)-mCherry expression in VTA. **G**, **H** Glucose tolerance of Vehicle+CNO, hM4D(Gi)+CNO (0 min: F_1,14_ = 0.631, *P* = 0.440; 15 min: F_1,14_ = 10.360, *P* = 0.006; 30 min: F_1,14_ = 9.699, *P* = 0.008; 60 min: F_1,14_ = 1.460, *P* = 0.247; 90 min: F_1,14_ = 0.097, *P* = 0.760; 120 min: F_1,14_ = 2.701, *P* = 0.123; n = 8). AUC, area under curves in glucose curve to present glucose tolerance (F_1,14_ = 5.826, *P* = 0.030; n = 8). **I** Bilateral cannula placement of D1R and D2R antagonist microinjection in mPFC. **J**, **K** Glucose tolerance of D1R^-^, D2R^-^ and vehicle groups. AUC, area under curves in glucose curve to present glucose tolerance (0 min: F_2,34_ = 0.976, *P* = 0.387; 15 min: F_2,34_ = 1.479, *P* = 0.242; 30 min: F_2,34_ = 2.644, *P* = 0.0.086; 60 min: F_2,34_ = 4.209, *P* = 0.023; 90 min: F_2,34_ = 3.310, *P* = 0.049; 120 min: F_2,34_ = 5.241, *P* = 0.010; AUC: F_2,34_ = 3.796, *P* = 0.033; post hoc test: *P < 0.05; *n* = 10-14). Error bars, ±s.e.m.

Although multiple earlier publications have described the VTA-mPFC dopamine projection [8, 32], we confirmed and validated VTA dopamine neuron projection to mPFC in DAT-Cre mice at the beginning of the study. We injected Fluoro-Gold into mPFC bilaterally and observed Fluoro-Gold signals that overlapped with DA neurons (colabelled with tyrosine hydroxylase/TH) in the VTA 1 week after injection (Fig S1a, b). To explore whether mPFC dopamine is involved in the mechanisms of glucose metabolism abnormality, we injected AAV2/9-DIO-eNpHR3.0-mCherry or AAV2/9-DIO-mCherry into the VTA of DAT-Cre mice as NpHR group or Vehicle control group (Fig. 1A, B). Direct light-triggered inhibition of neuronal firing was achieved by the expression of halorhodopsin (eNpHR3.0) in the NpHR group. Anatomical quantification showed NpHR expression in VTA cells projecting to mPFC became sparse but clear dopamine axons were nevertheless detected in the mPFC (Fig. 1B). Mice were given intraperitoneal glucose tolerance tests (IGTT) to examine glucose metabolism regulation. We found that optogenetically stimulated NpHR group had impaired glucose tolerance compared with stimulated vehicle group (Fig. 1C). Meanwhile, stimulated NpHR group had more abnormal glucose than unstimulated ones (Fig. S1d). We also observed the change of fasting glucose when NpHR and vehicle groups were stimulated by yellow light. However, both groups had no effect on the fasting glucose (Fig. S1c). Besides, there was no difference among fasting plasma insulin of stimulated NpHR group and vehicle group (Fig. S1e).

### Chemogenetic inhibition of VTA-mPFC dopamine projection caused glucose intolerance in mice

To further confirmed the role of VTA-mPFC dopamine projection in glucose metabolism, we used Designer Receptors Exclusively Activated by Designer Drugs (DREADD)-based chemogenetic tools. We injected AAV2/retro pAAV-TH-Cre-P2A-EGFP-WPRE into the mPFC and expressed DIO-hM4D(Gi)-mCherry or DIO-mCherry in the VTA as shown in Fig. 1E. In glucose tolerance test, we found disrupted glucose tolerance in hM4D(Gi) group compared with Vehicle group after the injection of CNO (Fig. 1G, H). Consistent with the results of optogenetic inhibition, both groups had no effect on the fasting glucose after intraperitoneal administration of CNO (Fig. S2b). However, we observed no difference in behavior tests associated with negative symptoms during chemogenetic inhibition and optogenetic inhibition, which was detected via tail suspension test and three-chambered test (Fig. S4a-d).

### Inhibition of D1Rs and D2Rs in mPFC causes glucose intolerance in mice

To further determine the role of prefrontal D1R or D2R in glucose tolerance, we locally infused vehicle (DMSO+saline), D1R antagonist, or D2R antagonist into mPFC of C57 mice. The infusion locations are shown in Fig. 1I and Fig. S2c. We found D2R antagonist infusion significantly impaired glucose tolerance while D1R antagonist had a slight disrupted effect on glucose tolerance (Fig. 1J, K). The inhibition of D2Rs had increased plasma glucose at 60 min, 120 min. And the inhibition of D1Rs had increased plasma glucose at 120 min. But only the inhibition of D2R decreased AUC in glucose tolerance test. Both D1R and D2R antagonists had no effect on the fasting glucose (Fig. S2d). Our findings confirm the effects of prefrontal cortical dopamine on the glucose regulation. Of note, the block of D2R instead of D1R increased the immobility in tail suspension test (Fig. S4e), but both exerted no effect on social behavior in three-chambered test (Fig. S4f-g).

### Disc1_tr_ mice show both impaired glucose metabolism and dopaminergic disturbances in prefrontal cortex

**Fig. 2:**
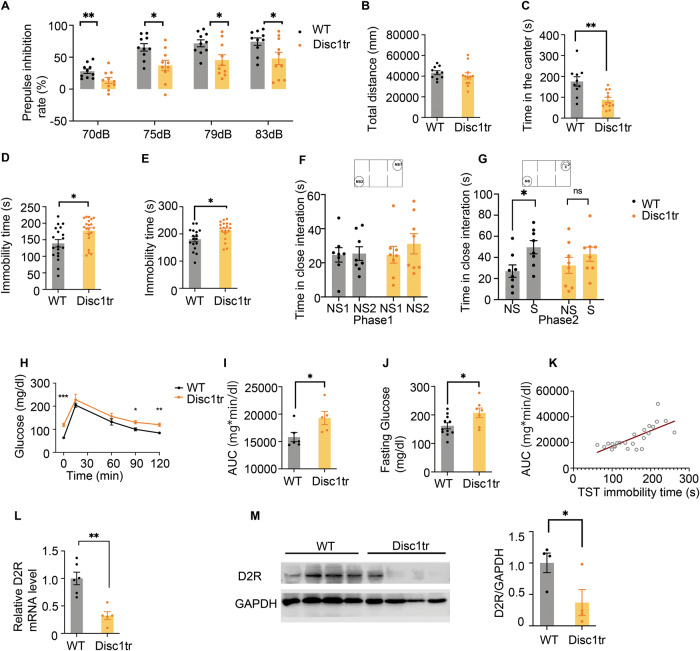
Impaired glucose metabolism and disrupted D2R expression in Disc1_tr_ mice. **A** Percentage of prepulse inhibition of the auditory startle reflex in four different prepulse intensities (70 dB, 75 dB, 79 dB, 83 dB) (70 dB: F_1,18_ = 6.348, *P* = 0.021; 75 dB: F_1,18_ = 8.123, *P* = 0.011; 79 dB: F_1,18_ = 6.937, *P* = 0.017; 83 dB: F_1,18_ = 5.450, *P* = 0.031; n = 10). **B** Total travel distance in open field test. No significant differences were observed (F_1,20_ = 0.739, *P* = 0.400; *n* = 10-12). **C** Exploration time in center area in open field test (F_1,20_ = 13.039, *P* = 0.002; *n* = 10–12). **D** Immobility in tail suspension test (F_1,37_ = 7.314, *P* = 0.010; *n* = 19–20). **E**, Immobility in force swim test (F_1,34_ = 7.366, *P* = 0.010, *n* = 17-19). **F**, **G**, Top panel, schematic diagram of three-chamber test, **F** Time in close interaction in Phase 1 for familiarization, no difference between non-social stimulus NS1 and NS2. **G** Time in close interaction when exposed to stranger mice (S) compare with non-social stimulus/NS (WT: F_1,14_ = 6.986, *P* = 0.019; Disc1_tr_: F_1,14_ = 1.087, *P* = 0.315; *n* = 8). **H**, **I** Glucose tolerance test for Disc1_tr_ mice (0 min: F_1.9_ = 37.073, *P* = 0.000182; 15 min: F_1.9_ = 0.971, *P* = 0.350; 60 min: F_1.9_ = 1.449, *P* = 0.259; 90 min: F_1,9_ = 10.034, *P* = 0.011; 120 min: F_1.9_ = 19.964, *P* = 0.002; *n* = 5–6). AUC, area under curves above baseline in glucose curve (F_1,9_ = 5.848, *P* = 0.039; *n* = 5–6). **J**, Fasting glucose of blood sample collected from heart was examined (F_1,16_ = 5.688, *P* = 0.030; *n* = 7–11). **K** The correlation of glucose tolerance AUC and tail suspension test (TST) immobility time (*r* = 0.727, *P* < 0.0001, *n* = 24). **L** Quantitative analysis of D2R mRNA expression in PFC (F_1,9_ = 22.433, *P* = 0.001, *n* = 5–6). **M**, Protein levels of D2R in the PFC (F_1,6_ = 6.155, *P* = 0.048; *n* = 4). Error bars, ±s.e.m.

We wondered whether impaired glucose metabolism existed in a classical animal model of schizophrenia and other psychiatric diseases, Disc1_tr_ mice. Disrupted-in-Schizophrenia-1 (*DISC1*) is a risk gene in many psychiatric diseases including schizophrenia. Our truncated DISC1 (Disc1_tr_) transgenic mice had impaired performance in prepulse inhibition (PPI) test of the acoustic startle response (Fig. 2A). This is indicative of the sensorimotor gating ability deficits, which are a characteristic of schizophrenia. Disc1_tr_ mice as reported previously [33, 34], exhibited longer immobility time in tail suspension test (Fig. 2D) and in forced swim test (Fig. 2E), which are considered as rodent depressive-like phenotypes. Disc1_tr_ mice exhibited anxiety-like symptoms as they spent less time in the center of chamber in a 20 min open field test (Fig. 2C). Disc1_tr_ mice also had impaired social behavior, showing no preference to a strange mouse compared with a non-social object in Phase 2 in three-chambered test (Fig. 2G). However, Disc1_tr_ mice had similar performance in anxiety-like elevated plus maze, light-dark box, and depression-like behavior sucrose preference to WT mice (Fig. S3a–c).

Interestingly, Disc1_tr_ mice had mild defects in glucose homeostasis regulation: impaired glucose tolerance, increased fasting glycemia (Fig. 2H–J). Of note, Disc1_tr_ mice had higher fasting glucose both in vivo and post-mortem. However, the Disc1_tr_ mice seemed to have intact peripheral insulin sensitivity because they had similar performance with WT littermate in insulin tolerance test (ITT) (Fig. S3e). To explore the association between glucose metabolism and negative symptoms in Disc1_tr_ mice, we conducted correlation analysis. As expected, there is a strong direct correlation between glucose tolerance AUC and tail suspension test (TST) immobility time (*r* = 0.727, *P* < 0.0001, *n* = 24) in Disc1_tr_ mice. Thus, Disc1_tr_ mice highly imitate the clinical phenomena we presented in the human cross-sectional study.

Although Disc1_tr_ mice had unchanged basal dopamine levels in PFC using post-mortem high-performance liquid chromatography with electrochemical detection (HPLC-ED) (Fig. S3d), Disc1_tr_ mice had a reduced expression of D2R expression in PFC via RT-PCR and western blot (Fig. 2L, M). In contrast, the TH and D1R expression in PFC were unchanged in Disc1_tr_ mice (Fig. S3i). Our findings of decreased D2R expression and abnormal glucose metabolism in Disc1_tr_ mice together similar findings in C57 mice after microinjection of D2R antagonists combine to underline the critical role of D2R-expressing neurons in mPFC. These neurons may be responsible to cause the abnormal glucose metabolism and negative symptoms in schizophrenia.

## Discussion and conclusions

In this series of human and rodent experiments, we show that systematic glucose metabolism, particularly glucose tolerance, may be regulated by the VTA-mPFC dopamine pathway in schizophrenia patients and animals. In the human cross-sectional study, we presented a robust positive association between glucose metabolism and negative symptoms in patients with schizophrenia. We show that iTBS improved fasting glucose metabolism and negative symptoms significantly. To further confirm the link between prefrontal cortical dopamine and glucose metabolism, we optogenetically and chemogenetically inhibited the VTA-mPFC dopamine pathway, and this led to subsequent glucose intolerance. In mPFC, D2R-expressing neurons are directly associated with glucose intolerance in mice revealed by micro-injection of dopamine receptor antagonists in the mPFC. Finally, Disc1_tr_ mice presented significantly impaired fasting glucose metabolism after starvation and decreased expression of D2Rs.

This is the first study that link the high-hierarchy brain area such as PFC with the regulation of systematic metabolism. In the previous studies, neurobiological underpinnings of the glucose homeostasis focused on the hypothalamus. Few studies had explored whether PFC, the central executive of the brain, takes part in the systematic glucose homeostasis. Of note, this study reported that VTA-mPFC dopamine pathway can regulate glucose tolerance in a direct and fast way.

The association between glucose metabolism and psychopathology in first-episode schizophrenia had been reported in many times [16, 17, 35, 36]. However, a robust association between impairment of glucose metabolism and negative symptoms has not been revealed yet. The most likely explanation is that our database of 704 drug naïve first episode schizophrenia is by far the largest group of drug-naïve first episode schizophrenia subjects examined to date. This has allowed us to identify and study as an independent subgroup a substantial cohort of drug-naïve patients (*n* = 147) with predominant negative symptoms. There are some limitations in this cross-sectional study, because the duration of symptoms is not strictly matched in the four groups (Positive, Negative, Positive & Negative group, Other patients). In previous studies, researchers used to ignore the effect of duration when investigating the first-episode psychosis. But in this study, we find these four groups had rather different duration, where Negative group had much longer duration than Positive group. The worse glucose metabolism in Negative group may result from the longer course. Thus, more well-controlled descriptive studies are in need to support this hypothesis.

The iTBS study targeting DLPFC confirmed its good efficacy to improve negative symptoms. As revealed via magnetic resonance imaging, pronounced negative symptoms in schizophrenia are associated with disrupted connectivity in DLPFC (only found in primates) [37] and mPFC [38]. Many TMS studies targeted DLPFC were revealed to improve negative symptoms in patients with schizophrenia [39]. Based on these observations, we have hypothesized that increased activity at some dopamine terminals in PFC may be responsible for the improvement in both negative symptoms as well in glucose metabolism. Although TBS had potential to activate the dopamine circuits, its application in metabolic disorders had not been explored. This study may widen the application of iTBS in metabolism disorder. Other physical interventions such as DBS, targeting to activate dopaminergic neurons, have had many applications in obesity and other metabolic disorders [40]. DBS targeting the striatum improved glucose metabolism via increasing insulin sensitivity [19]. In addition, transcranial direct current stimulation (tDCS) improves glucose tolerance via increasing brain energy consumption, which is an insulin-independent mechanism [41, 42]. We noticed that iTBS decreased both fasting glucose and fasting insulin, and the change of fasting insulin was positively correlated with the change of SANS score. These results indicated that iTBS is different from tDCS and might reduce blood glucose through an insulin-dependent mechanism.

To our knowledge, this study revealed that VTA-mPFC dopamine projection regulated systematic glucose for the first time. The defects of VTA-mPFC dopamine projection linked pathology of schizophrenia to glucose metabolism. However, we failed to detect any difference in fasting glucose or fasting insulin after inhibiting the dopamine projection. It remains unclear whether the glucose intolerance is insulin-dependent or not. Besides, the inhibition of VTA-mPFC dopamine projection failed to mimic negative symptoms in mice, which is consistent with Dipesh Chaudhury’s study. In their study, mice had intact social interaction if they were not previously undergone subthreshold social defeat [8].

Our results have also demonstrated that D2R-expressing neurons in mPFC play important roles on glucose regulation. As reported previously, there are two major subtypes of principal neurons in the mPFC: D2R/type A and D1R/type B. D2R/type A neurons with complex dendritic arborization are mainly in deep layers while D1R/type B neurons are distributed through deep and superficial layers. D2Rs and D1Rs may play different roles in regulating metabolism and negative symptoms. We found D2R-expressing neurons play the major role, but some studies had inconsistent conclusion that optogenetic activation of D1R-expressing neurons rather than D2R-expressing have long-term anti-depressant effect [43]. We assumed that firstly the long-term anti-depressant effect may be different with the instant depression-induced effect in this study, and secondly the optogenetic activation may have different effect from antagonist injection. Besides, D1R-expressing neurons in the mPFC regulated food intake [44]. The D2Rs in brain and peripheral organs have a critical role in glucose metabolism. For instance, intracerebroventricular injection of D2R antagonists or agonists induced the rise of plasma glucose immediately via autonomic nerves [45]. Chronic administration of D2R agonists improved systemic glucose metabolism [46]. Apart for D2Rs in brain, D2Rs in peripheral organs such as pancreas play important roles. For example, D2R antagonist administration regulated circadian rhythms and insulin secretion function of pancreas β cells [47, 48]. On the other hand, the interaction between D2R and DISC1 protein has been reported many times [49–51], which may account for our results that Disc1_tr_ mice had decreased D2R expression in PFC.

There remain some limitations in our studies. Firstly, no PET/CT data supported the relationship between the dopamine in PFC and glucose metabolism in the iTBS human study. The stimulating targets of iTBS may be inconsistent with the activating brain areas. Thus, for further exploration, it is necessary to involve PET dopamine imaging using D2R radioligand in iTBS study. Secondly, the downstream neuronal circuits of D2R-expressing neurons regulating glucose tolerance remain to be elucidated. Thirdly, it is necessary to detect the change of some circulating stress hormones and perform hyperinsulinemic-euglycemic clamps to explore the exact mechanism of glucose regulation.

In conclusion, our results demonstrate that VTA-mPFC dopamine projections and D2R-expressing neurons may regulate glucose tolerance in schizophrenia.

### Supplementary information

Supplementary materials

## Data Availability

All data required to evaluate the conclusions are exhibited in the manuscript and/or the Supplementary Materials. These original data are available from the corresponding author upon reasonable request.
