# Supplementary material for: Prefrontal cortical dopamine deficit may cause impaired glucose metabolism in schizophrenia
Source: Transl Psychiatry. 2024 Feb 6;14:79. doi: 10.1038/s41398-024-02800-7 (PMC10847097; doi:10.1038/s41398-024-02800-7)
Supplement: Supplementary file 1 — Supplementary materials [file 41398_2024_2800_MOESM1_ESM.docx]

**SUPPLEMENTARY MATERIALS**

**Table S1. Descriptive results of 704 first-episode patients with schizophrenia**

|  | Mean (SD) | Median | Min | Max |
| --- | --- | --- | --- | --- |
| Age (years) | 27.44 (8.91) | 26 | 10 | 69 |
| Duration of illness (months) | 14.82 (12.99) | 12 | 1 | 60 |
| Height (cm) | 165.40 (8.04) | 165 | 145 | 195 |
| Weight (kg) | 59.31 (12.33) | 57.35 | 32.50 | 125.60 |
| BMI (kg/m2) | 20.67 (6.07) | 21.00 | 9.63 | 49.68 |
|  |  |  |  |  |
| PANSS |  |  |  |  |
| Positive score | 20.81 (6.77) | 20 | 7 | 44 |
| Negative score | 20.75 (7.38) | 21 | 7 | 44 |
| General psychopathology | 40.31 (9.43) | 39 | 16 | 92 |
| Total score | 81.82 (17.43) | 80 | 34 | 155 |
|  |  |  |  |  |
| FG (mmol/L) | 5.12 (0.94) | 4.90 | 3.20 | 8.50 |
| FI (mU/L) | 16.51 (11.14) | 13.65 | 1.57 | 72.33 |
| HOMA-IR | 3.90 (3.06) | 3.04 | 0.28 | 24.43 |
|  |  |  |  |  |
| TG (mmol/L) | 1.27 (1.01) | 0.97 | 0.22 | 9.49 |
| Chol (mmol/L) | 4.16 (1.79) | 4.07 | 0.32 | 31.50 |
| HDL-C (mmol/L) | 1.30 (0.47) | 1.23 | 0.62 | 5.56 |
| LDL-C (mmol/L) | 2.43 (0.69) | 2.38 | 0.75 | 4.94 |

Abbreviations: FG, fasting glucose; FI, fasting insulin; TG, Triglyceride; Chol, Total cholesterol; HDL-C, High-density lipoprotein cholesterol; LDL-C, Low-density lipoprotein cholesterol; PANSS, Positive and Negative Syndrome Scale.

**Table S2. The baseline characteristics of patients with schizophrenia in iTBS study**

|  | iTBS (n=28) | Control (n=29) | *P* value |
| --- | --- | --- | --- |
| Age | 26.72 (7.87) | 26.59 (6.75) | 0.924 |
| Gender (female, male) | (15,13) | (13,16) | 0.509 |
| Duration of illness (year) | 4.70 (2.26) | 4.18 (1.56) | 0.227 |
|  |  |  |  |
| PANSS Positive | 13.39 (2.36) | 13.31 (2.58) | 0.075 |
| PANSS Negative | 28.32 (2.47) | 27.55 (2.78) | 0.102 |
| PANSS General | 37.68 (6.32) | 37.48 (5.39) | 0.902 |
| PANSS Total | 78.39 (7.67) | 78.34 (7.36) | 0.607 |
| SANS | 68.32 (11.84) | 66.38 (7.12) | 0.829 |
|  |  |  |  |
| FG (mmol/L) | 5.52 (0.84) | 5.19 (0.79) | 0.075 |
| FI (mU/L) | 13.28 (7.55) | 12.40 (6.86) | 0.609 |
| HOMA-IR | 3.34 (2.14) | 2.91 (1.89) | 0.303 |

Values are presented as mean (SD).

Abbreviations: PANSS, Positive and Negative Syndrome Scale; SANS, Scale for the Assessment of Negative Symptoms; FG, fasting glucose; FI, fasting insulin.

iTBS group underwent active iTBS while Control group underwent sham stimulation.

**Table S3. Correlation Test Matrices at baseline in participants.**


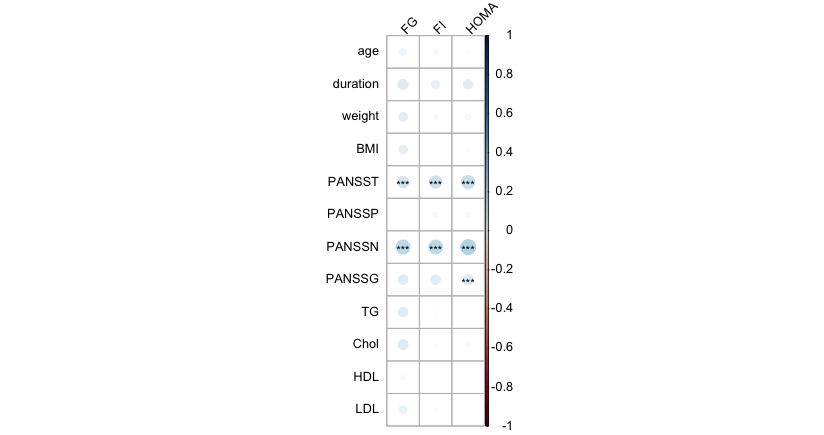


Abbreviations: PANSST, the total score of Positive and Negative Syndrome Scale; PANSSP, the score in positive symptom subscale; PANSSN, the score in nagetive symptom subscale; PANSSG, the score in general psychopathology subscale; FG, fasting glucose; FI, fasting insulin; TG, Triglyceride; Chol, Total cholesterol; HDL-C, High-density lipoprotein cholesterol; LDL-C, Low-density lipoprotein cholesterol.

**Table S4. Participant flow**

## Intervention

Excluded (n=24)

♦  PANSS-N less than 24 (n=11 )

♦  Unstable medication (n=10)

♦  Declined to participate (n=5 )

♦  Other reasons (n= 2)

Assigned (n=60)

Assessed for eligibility (n=84)

Analysed (n= 29)
♦ Excluded from analysis (n=1)

Analysed (n=28)
♦ Excluded from analysis (n=2)

Finished intervention (n=29)

Withdraw informed consent (n=1)

Allocated to Control (n= 30)

♦ Received allocated intervention (n=30)

## Analysis

## Allocation

## Enrollment

**
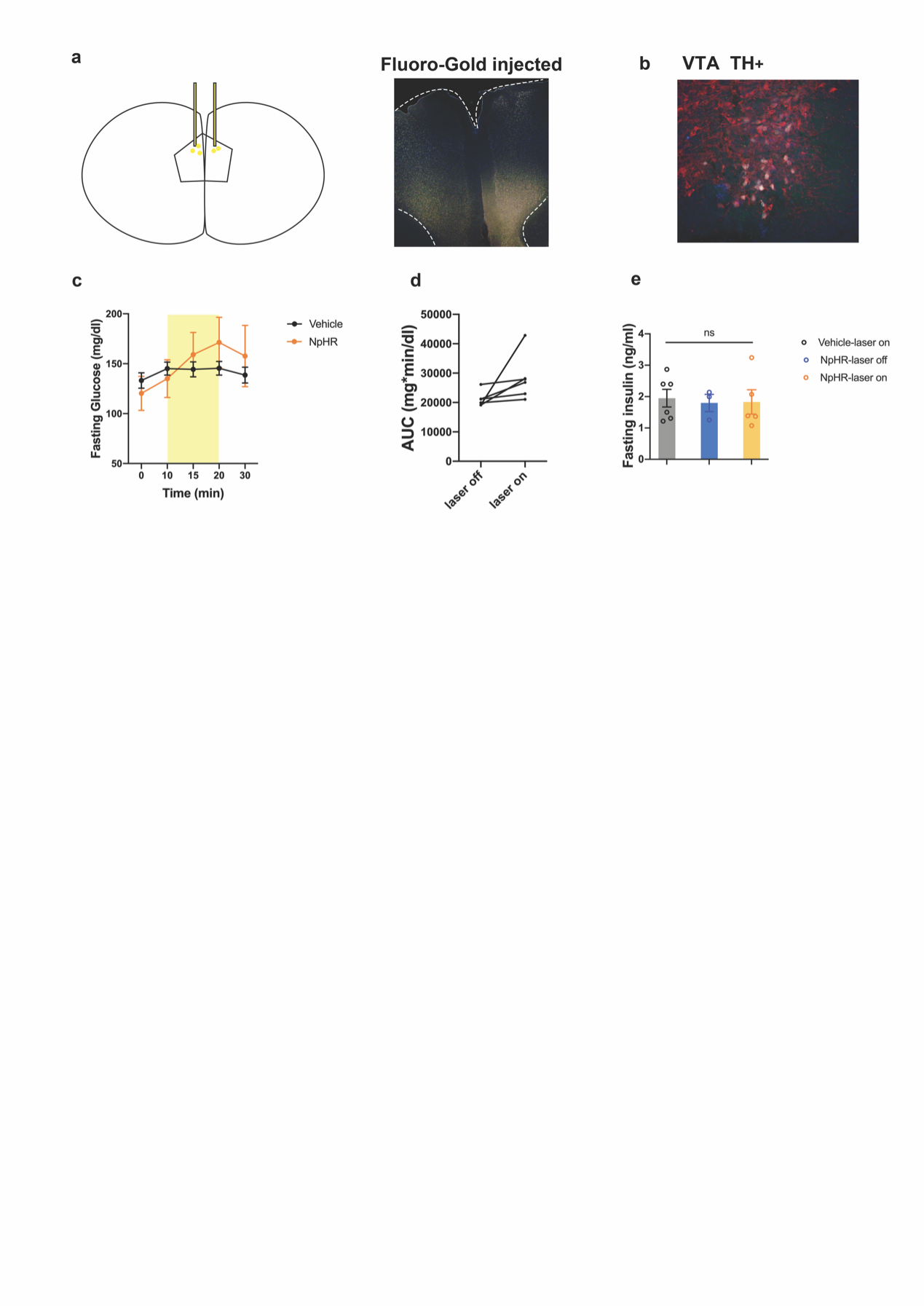
**

**Fig.S1 The effects of optogenetic inhibition of VTA-mPFC dopamine projection on glucose metabolism. a,** Fluoro-Gold injected into mPFC. b, confocal images of VTA shows co-expression of Fluoro-Gold in TH-positive cells. **c,** The change of fasting glucose after the stimulation and the yellow panel was in the laser-on phase. (0min; F_1,8_=0.479, *P*=0.694; 10min: F_1,8_=0.254, *P*=0.628; 15min: F_1,8_=0.396, *P*=0.546; 20min: F_1,8_=0.984, *P*=0.350; 30min: F_1,8_=0.363, *P*=0.564; n=5). **d,** the area under curves (AUC) of glucose curve in tolerance test from laser-on and laser-off NpHR mice. e, fasting insulin of blood sample collected from heart from vehicle+ laser-on, NpHR+laser off, NpHR+laser on (F_2,11_ =0.058, *P*=0.944; n=3-6). Error bars, ±s.e.m.


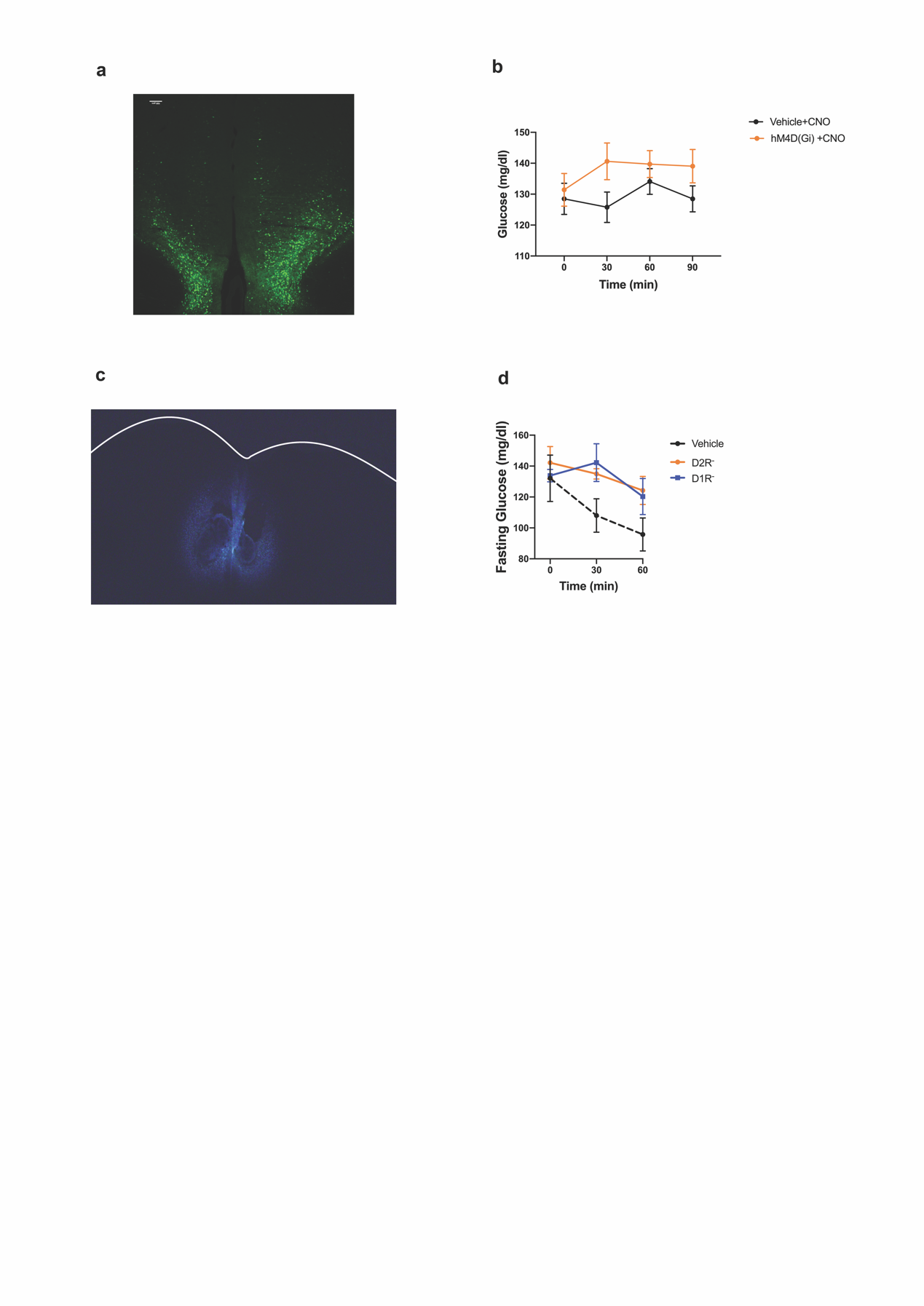


**Fig.S2 Chemogenetic inhibition of VTA-mPFC, and microinjection of D1/D2R antagonists in mPFC.** **a,** Bilateral expression of AAV2/retro pAAV-TH-Cre in mPFC. **b,** Fasting glucose 0min, 30min, 60min, 90min after CNO injection**.** (0min; F_1,14_=0.161, *P*=0.694; 30min: F_1,14_=3.709, *P*=0.075; 60min: F_1,14_=0.874, *P*=0.366; 90min: F_1,14_=2.394, *P*=0.144; n=8). **c,** Bilateral cannula placement after microinjection of Fluoro-Gold, **d,** Fasting glucose 0min, 30min, 60min after microinjection. (0min; F_2,13_=0.267, *P*=0.770; 30min: F_2,13_=3.144, *P*=0.077; 60min: F_2,13_=1.967, *P*=0.179; n=5-6). Error bars, ±s.e.m.





**Fig.S3 Behavior tests, glucose metabolism of Disc1_tr_ mice. a,** Open arm exploration time in elevated plus maze (F_1,18_=1.055, *P*=0.318; n=10). **b,** Exploration rate of light box in light-dark box (F_1,14_=1.198, *P*=0.292; n=8). **c,** Preference in sucrose preference test (F_1,14_=166, *P*=0.690; n=8). **d,** Total dopamine level in PFC via post-mortem high-performance liquid chromatography with electrochemical detection. (F_1,14_=0.085, *P*=0.775; n=8) **e-f,** insulin tolerance test. AUC, area under curves (0min: F_1,23_=0.488, *P*=0.492; 30min: F_1,23_=2.709, *P*=0.113; 60min: F_1,23_=0.222, *P*=0.642; 60min: F_1,23_=0.579, *P*=0.454; AUC: F_1,23_=1.009, *P*=0.326; n=11-14). **g-h,** plasma insulin of blood sample collected from tail vein at overnight starvation and 15min after glucose injection (F_1,15_==0.439, *P*=0.517; n=8-9).  **i,** TH and D1R level in PFC. Error bars, ±s.e.m.


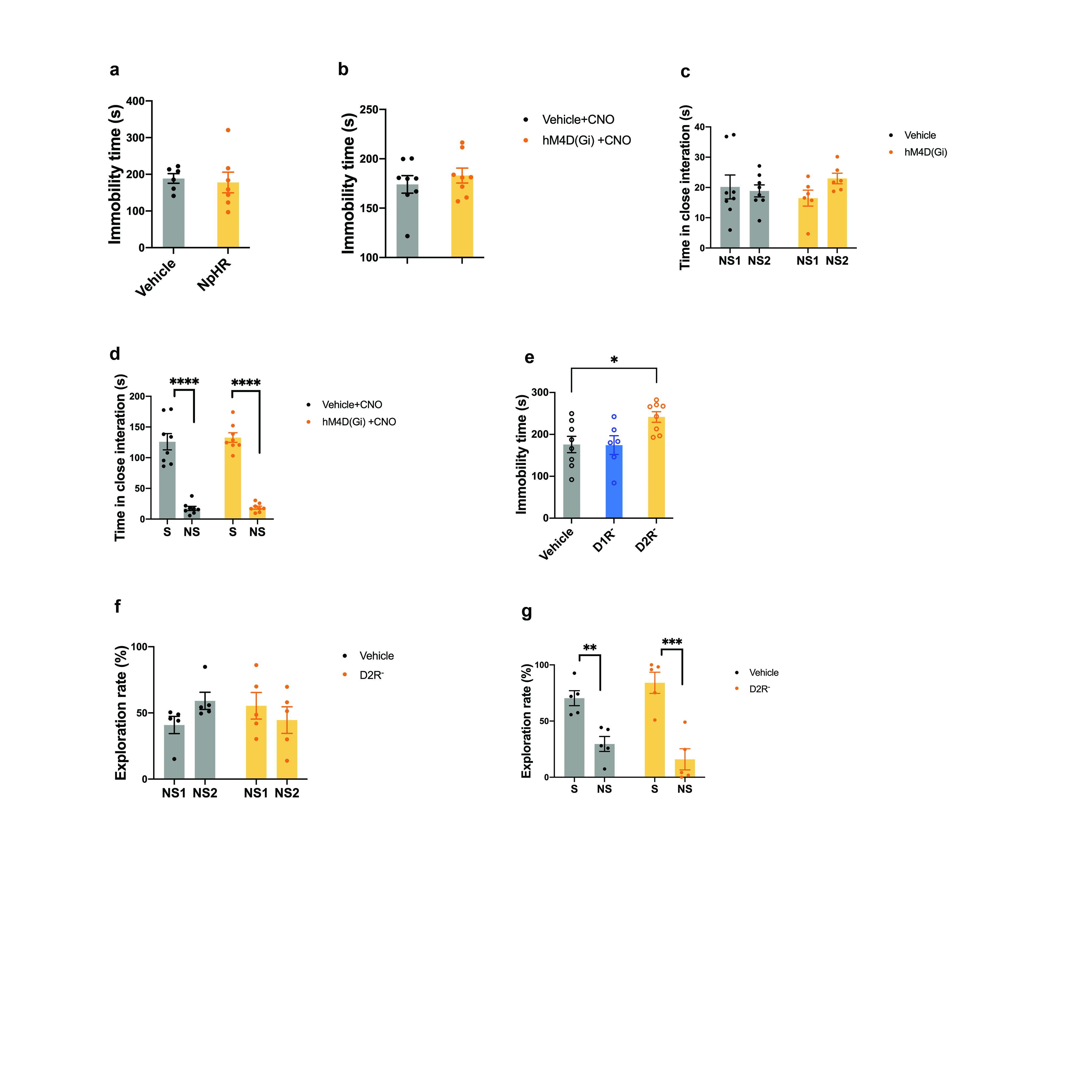


**Fig.S4 Depressive-like behavior tests during optogenetic inhibition, chemogenetic inhibition and dopamine antagonist injection. a,** Immobility of tail suspension test during optogenetic inhibition. (F_1,11_=0.110, *P*=0.746, n=6-7). **b,** Immobility in tail suspension test during chemogenetic inhibition. (F_1,14_=0.586, *P*=0.457, n=8). **c-d,** Three-chambered test during chemogenetic inhibition. **c,** The familiarization session. **d,** The test session. **e,** Immobility of tail suspension test after microinjection of dopamine receptor antagonists. (F_2,19_=4.711, *P*=0.022; n=6-8, post hoc test, **P*<0.05). **f-g,** Three chambered test after microinjecting of D2R antagonist. **f,** The familiarization session**. g,** The test session.
